# An Analysis of Noise in Multi-Bit ΣΔ Modulators with Low-Frequency Input Signals

**DOI:** 10.3390/s22197458

**Published:** 2022-10-01

**Authors:** Pablo Vera, Andreas Wiesbauer, Susana Paton

**Affiliations:** 1 Electronics Technology Department, Carlos III University, 28903 Madrid, Spain; 2 Infineon Technologies Austria, 9500 Villach, Austria

**Keywords:** ΣΔ, ADC, multi-bit, noise, DAC, mismatch, linearity, quantization noise, estimation, prediction

## Abstract

Digital and smart sensors are commonly implemented using multi-bit ΣΔ Modulators. Undesired signals can be present at the ADC input, such as low-frequency signals with medium or high amplitude, as a consequence of mechanical artifacts in the MEMS and/or temporary signal overload. Simulations and measurements of those sensors with such signals show temporary increments of in-band noise power. This paper investigates the factors that produce this transient performance loss. Interestingly, noise increments happen when the modulator is forced to toggle between three adjacent levels and is not correlated with the typical tonal behavior of ΣΔ Modulators. Hence, the sensor performance is sensitive to some specific input patterns even if tonal behavior is decreased by dithering the input of the ADC. Different error sources, such as the mismatch between DAC cells, loop filter linearity error, and quantization error, contribute to the observed noise increments. Our aim is to analyze each of these error sources to understand and quantify in-band noise power increments, and to desensitize the ADC from the undesired input patterns. Some estimation equations are proposed and verified through extensive simulations, by means of deterministic and stochastic methods. These equations are influenced by some modulator parameters and can be used to optimize them in order to reduce such in-band noise power increments.

## 1. Introduction

The increase in the noise power at the output of the Analog-to-Digital Converter (ADC) has been experimentally observed in some digital and smart sensors under certain input patterns, such as undesired low-frequency signals (lower than 1 Hz) or DC. Many integrated sensors require an ADC with a resolution higher than 15 bits, low bandwidth (less than 100 kHz) and low power consumption. Discrete time (DT) multi-bit Sigma-Delta Modulators (ΣΔM) are an excellent choice for these applications [1,2,3,4]. The sensor performance can be degraded when certain input patterns are applied because the In-Band Noise (IBN) power of the DT-ΣΔM is increased. This behavior could be difficult to measure while being tested in a laboratory because it cannot be detected using typical measurement setups, such as idle channel or medium-frequency (1 kHz) sinusoidal signals. The only way to measure it properly is to apply a very slow ramp at the modulator input and split the output signal into small segments. The IBN power of each segment can be calculated. Then, a direct correlation between the input amplitude and the IBN power can be established. A pre-silicon analysis is strongly required because it allows the detection of noise bumps during the design phase. This analysis is also useful to optimize some system-level design parameters in the ΣΔM.

**Figure 1:**
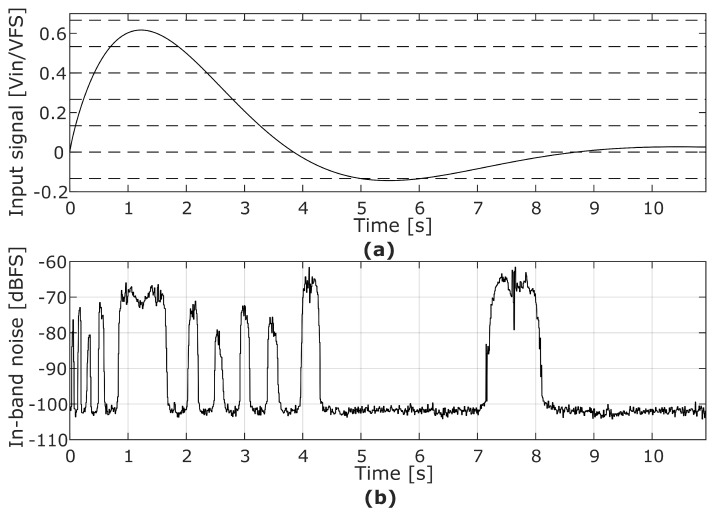
(**a**) Slow input signal caused by a hit (solid line) and quantizer thresholds (dashed horizontal lines). (**b**) In−band noise power.

**Figure 2:**
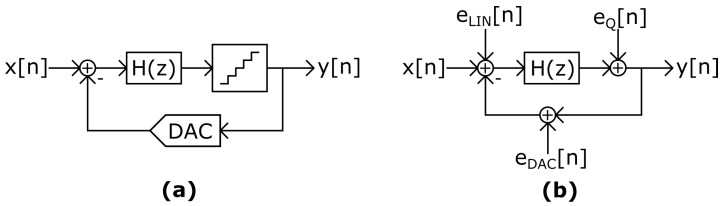
(**a**) System-level model of a DT-ΣΔM. (**b**) Linear model of IBN power increase in DT-ΣΔM with low-frequency input signals.

A simulation of this phenomenon can be seen in Figure 1. This input pattern may happen in a microphone being tapped by the user. A high and loud pressure is created by a tap or a small shock event that saturates the MEMS sensor. The signal is slowly restored while the MEMS sensor is being discharged, and the ΣΔM shows intermittent IBN bumps at its output that are audible on top of the captured sound. The starting point for this analysis is the observations from simulations and measurements in multi-bit ΣΔM based sensors. Figure 2a shows the system-level model of a ΣΔM, including the loop filter H(z), the flash quantizer and the feedback DAC. Noise bumps are a direct consequence of the input pattern. The input signal slowly crosses the quantizer thresholds if it has a low-frequency component with medium or high amplitude. When the input amplitude is between two adjacent quantizer thresholds, it forces the feedback Digital-to-Analog Converter (DAC) to use three adjacent levels, increasing the IBN power. As seen in Figure 2b, three main contributors to these noise bumps have been identified in this situation: mismatch between DAC cells ($e_{DAC}$), loop filter linearity error ($e_{LIN}$), and quantization error ($e_{Q}$), being the first two the main contributors. These error sources are widely known [1], but their contribution to the observed noise bumps has not been analyzed yet. Mismatch and linearity errors are consequences of circuit non-idealities, while quantization error would produce such noise bumps even in a completely ideal ΣΔM. The main goal of this manuscript is the analysis and quantification of those identified noise sources in increased noise power regions, as observed in Figure 2. This is useful to optimize the design.

This paper is organized as follows. Section 2 reviews the related literature and analyzes the origin of the observed IBN bumps. Section 3 studies the effect of DAC mismatch, loop filter linearity error, and quantization noise on IBN power increase. Deterministic and stochastic methods to predict the noise power increase are proposed. Section 4 compares the contribution of the different error sources and proposes some guidelines to optimize the modulator design parameters. Finally, Section 5 concludes the article.

## 2. Materials and Methods

### 2.1. Related Work

In this section, we provide a survey of the main studies of the output pattern of ΣΔM. Behavior and stability of single-bit ΣΔM with DC inputs have been studied in the literature, focusing on the first-order case [1,5]. It has been shown that DC input signals create idle tones at the output spectrum, whose power and frequency depend on the input DC signal [1]. Single-bit second and third-order ΣΔM stability has been analyzed in [6], obtaining bounds on the loop gains for stable operation. The output signal bit pattern is characterized and the statistical properties of the quantization noise are analyzed for single-bit high-order ΣΔM in [7]. An accurate analysis of the basic second-order 2-bit ΣΔM based on its DT model is presented in [8]. Circuit non-idealities effects in switched capacitor ΣΔMs, such as amplifiers finite gain, dynamic limitations and gain non-linearity, comparators hysteresis, and mismatch have been widely addressed and reported [1,9].

To the best of the authors’ knowledge, the observed noise power increase in multi-bit high-order ΣΔM with low-frequency input signals has not been analyzed and reported in the literature.

### 2.2. Origin of Increased Noise Power in the Presence of Slow Input Signals

The analysis of this work is focused on low-frequency (lower than 1 Hz) input signals; a similar behavior as the observed with DC signals (0 Hz) might be expected. Hence, we consider in this section several potential contributors: idle tones, as described in the related literature for DC inputs, quantization noise as usually considered in the linear model, and circuit related errors as mismatch and linearity errors (see Figure 2). In-band noise power is obtained by extensive transient simulation of the aforementioned contributors. Example ΣΔMs, as depicted in Figure 2, are used along the article. They are generated with the Schreier toolbox [10], with maximally flat Noise Transfer Function (NTF) and optimized zeros. According to Figure 2, the NTF and Signal Transfer Function (STF) are defined as follows:

(1) $$Y\left(z\right)=X\left(z\right)\cdot \mathrm{STF}\left(z\right)+E_{Q}\left(z\right)\cdot \mathrm{NTF}\left(z\right)$$

(2) $$\mathrm{NTF}\left(z\right)=\frac{1}{1+H(z)}$$

(3) $$\mathrm{STF}\left(z\right)=\frac{H(z)}{1+H(z)}.$$

**Figure 3:**
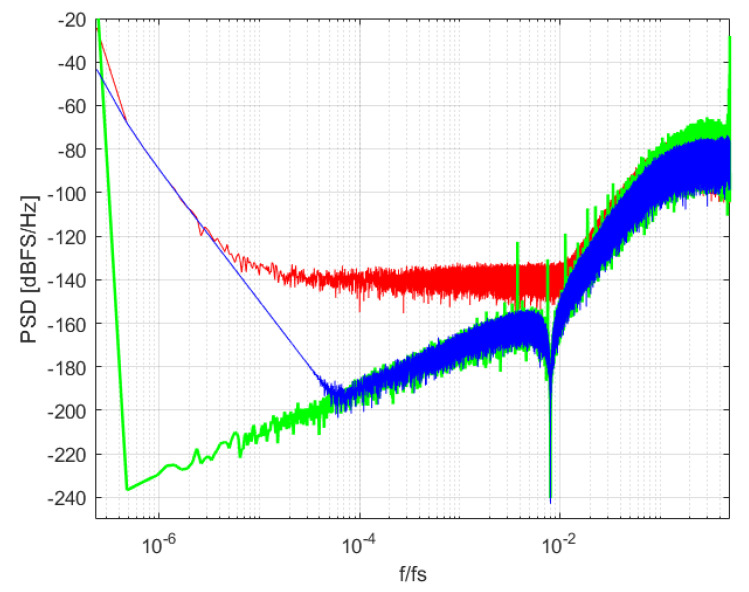
PSD comparison of low IBN power (blue), high IBN power (red), and DC input of 4 kHz (green). $2^{24}$ points (8.73 s) are simulated. ΣΔM parameters are: third-order, 4-bits, BW=20 kHz, OSR=48, OBG=1.8.

Idle tones in ΣΔMs are well known and have been reported in the literature [1,11]. DC signals produce a periodic output pattern; this behavior creates idle tones in the output sequence. Idle tones frequency and amplitude depend on the input DC level, the modulator parameters, and the number of bits of the quantizer. These tones can lie inside or outside of the modulator bandwidth. If these tones are in the modulator bandwidth, IBN power is increased. Idle tones outside of the modulator bandwidth are usually removed by the decimation filter at the modulator output. The Power Spectral Density (PSD) of the output signal of an example ΣΔM with a constant input of 0.4 V can be seen in the green graph of Figure 3. Idle tones in and outside of the modulator bandwidth can be observed. This is compared with the output of the same ΣΔM when a very slow ramp is applied. The third-order modulator has an OverSampling Ratio (OSR) of 48, a bandwidth of 20 kHz an its NTF has an Out-of-Band-Gain (OBG) of 1.8. The following transfer functions have been obtained with the Schreier toolbox [10]:

(4) $$H\left(z\right)=\frac{1.141z^{-1}-1.722z^{-2}+0.6918z^{-3}}{1-2.999z^{-1}+2.999z^{-2}-z^{-3}}$$

(5) $$\mathrm{NTF}\left(z\right)=\frac{1-2.999z^{-1}+2.999z^{-2}-z^{-3}}{1-1.858z^{-1}+1.277z^{-2}-0.308z^{-3}}$$

(6) $$\mathrm{STF}\left(z\right)=\frac{1.141z^{-1}-1.722z^{-2}+0.6918z^{-3}}{1-1.858z^{-1}+1.277z^{-2}-0.308z^{-3}}.$$

**Figure 4:**
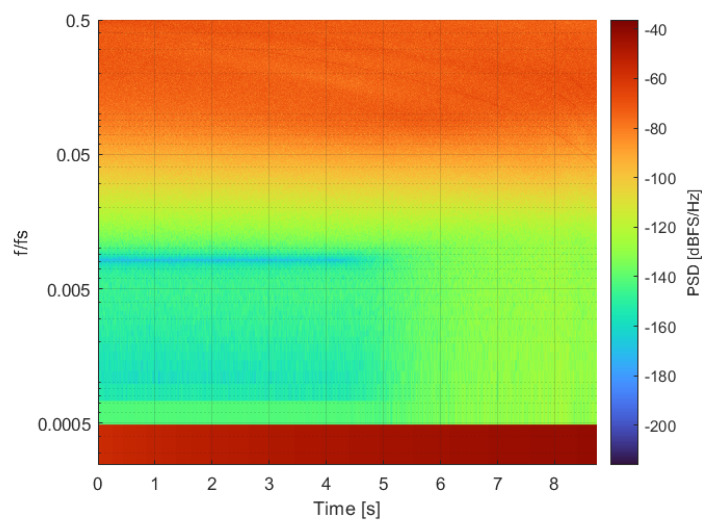
Spectrogram of the ΣΔM output; the signal is divided into 8192 segments of 1.06 ms to compute the spectrogram. Input signal is a ramp that starts at 0.01 V and ends at 0.06 V. Modulator parameters are: third-order, 4-bits, BW=20 kHz, OSR=48, OBG=1.8.

Experiments show that the IBN bumps observed in Figure 1 are not created by idle tones; IBN power is increased because the noise floor is increased when the modulator works under certain conditions. This behavior can be observed in the spectrogram shown in Figure 4. The input signal is a very slow ramp that starts at 0.01 V and ends at 0.06 V. Two different regions (separated by the time instant at 5 s) can be observed in the spectrogram: the first one has low IBN power, and the second one has high IBN power. The first region of the spectrogram is composed of the input signal component (low frequencies), filtered quantization noise (the notch of the NTF can be seen at 15.36 kHz), and high frequency quantization noise. The PSD in this region can be seen in the blue trace of Figure 3. The ΣΔM uses two different DAC levels in this region. The second region is composed by the input signal component (low frequencies), white noise in the modulator bandwidth, and high-frequency quantization noise. The PSD in this region can be seen in the red trace of Figure 3. The ΣΔM uses three different DAC levels in this region. This comparison proves that increased IBN power with low-frequency input signals is caused by increased noise floor, not by idle tones.

**Figure 5:**
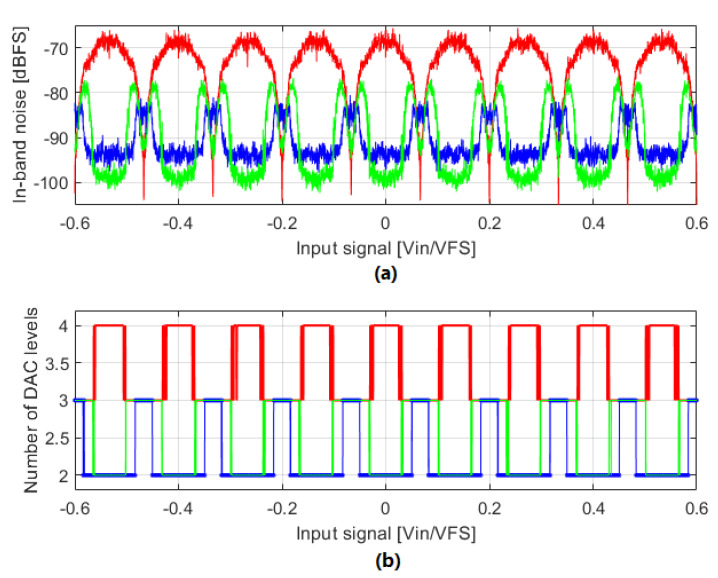
(**a**) IBN power and (**b**) number of used DAC levels. ΣΔM common parameters are: third-order, 4-bits, OSR=32, BW=20 kHz. OBG values are 1.5 (blue), 1.8 (green), and 2.5 (red). Input signal is a slow ramp whose slope is 91.5 mV/s. Output signal is divided in segments of 3.2 ms. $e_{LIN}$ and $e_{Q}$ are included.

From the experiments described in Figure 3 and Figure 4, it is observed that the noise sources shown in Figure 2 increase IBN power when more than two different DAC levels are used. These noise sources effect are analyzed in the following sections. The number of used DAC levels is determined by the modulator NTF and the input signal. NTF aggressiveness can be defined by its OBG. This parameter represents the constant gain of the NTF at high frequencies [1]. High order and aggressive NTFs increase the activity of the quantizer input signal, rise its swing and consequently the probability of using more than two levels. If the NTF is very aggressive, the modulator can be forced to always use more than two DAC levels independently of the input signal. In this case, IBN power is almost always increased by the noise sources shown in Figure 2. This case offers very high IBN power. Three modulators with different OBG have been simulated in Figure 5. The NTFs of these modulators have been designed according to the criteria explained in [1], and using the Schreier toolbox [10]. As a short summary, the resolution of a ΣΔM can be improved by increasing the modulator order, the OBG, the OSR or the number of bits. A simulation example with OBG=2.5 can be seen in the red trace of Figure 5. This case forces the use of more than two levels for all the stable input amplitudes.

NTFs with moderate OBG values do not force the modulator to always use more than two levels. Depending on the input signal value, it can force the modulator to use more than two DAC levels. Two simulation examples can be seen in blue (OBG=1.5) and green (OBG=1.8) traces of Figure 5. IBN power is lower when only two DAC levels are used; in these regions, the overall noise power is dominated by the quantization noise. NTFs with high OBG have reduced IBN power in these regions because the NTF has better filtering capabilities. When the input signal is between two adjacent quantization thresholds, it forces the modulator to use more than two levels. IBN power is increased in these regions; the higher the OBG, the higher the IBN power. On top of that, the increased noise regions are wider for higher OBG values. Let us define the regions in which the input amplitude is between two adjacent quantizer thresholds as Noise Prone (NP) regions. There are $2^{Nbits}-2$ NP regions, where Nbits is the number of bits in the quantizer. Mid-rise quantizers are used in this article. Least-significant bit voltage ($V_{LSB}$) is defined in (7), where $V_{FS}$ represents the quantizer full-scale voltage. The location of the NP regions is defined in (8).

(7) $$V_{LSB}=\frac{V_{FS}}{2^{Nbits}-1}$$

(8) $$NP\left[k\right]=\left(-2^{Nbits-1}+k+0.5\right)\cdot V_{LSB},\;\;\;k=1,2,\ldots ,2^{Nbits}-2.$$

#### Influence of Dither

**Figure 6:**
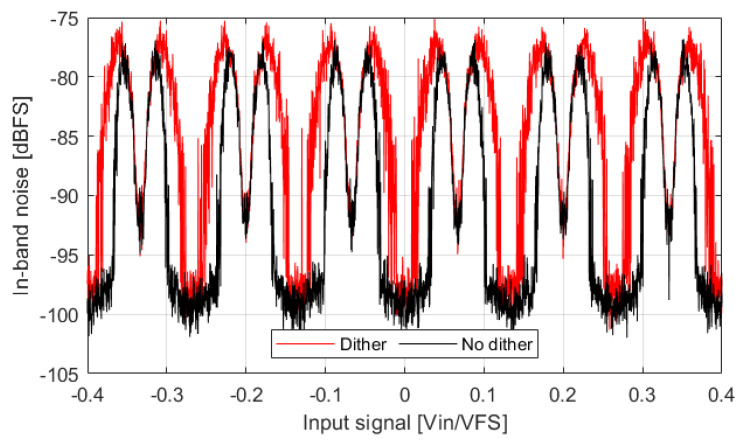
IBN power of a ΣΔM whose parameters are: third-order, 4-bits, OSR=32, BW=20 kHz, OBG=1.8. Output signal is divided into segments of 3.2 ms.

The effect of dither on IBN power bumps is also analyzed to find out whether it can help to reduce them. Dither injects a pseudorandom sequence at the quantizer input that helps to decorrelate the quantization error and the input signal; it has been widely used to attenuate idle tones [12,13,14]. Dither has been implemented with a thirteenth-order Linear Feedback Shift Register (LFSR). The signal activity at the quantizer input is increased and the probability of using more than two levels is increased. Figure 6 shows the effect of dither on IBN power. It is clear that NP regions are wider and noise power is slightly higher. Dither does not decrease IBN power in or outside of NP regions. Both lowpass and highpass dither create the same effect as unfiltered dither. We tried to achieve constant IBN power by forcing the ΣΔM to use three DAC levels for all the input signals; this is not possible (even if the dither amplitude is manually optimized), and noise bumps are always present. The results lead to the conclusion that dither is not appropriate to reduce the observed IBN bumps.

## 3. Evaluation of Noise Increase

### 3.1. The Effect of DAC Mismatch on IBN Power Increase

**Figure 7:**
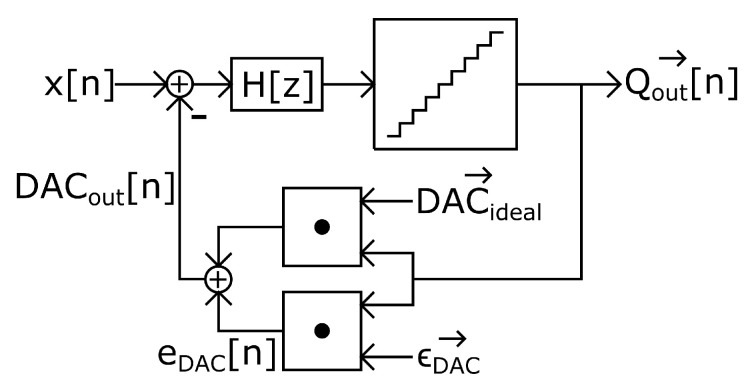
System-level model of a DT-ΣΔM showing how the DAC mismatch introduces an additional error.

**Figure 8:**
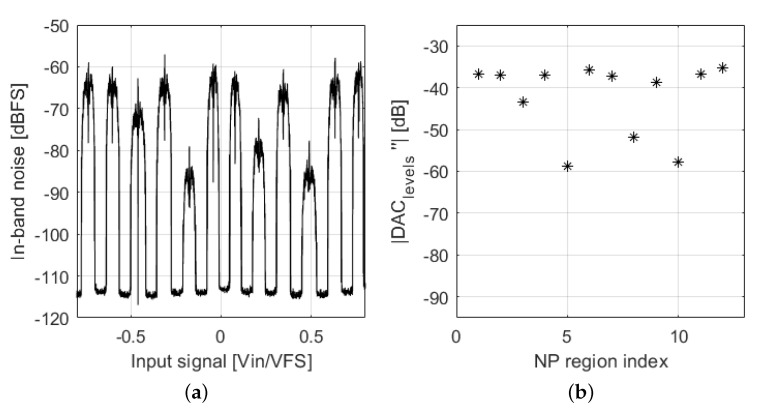
(**a**) In−band noise power; output signal is divided in segments of 4.8 ms. (**b**) $|DAC_{levels}^{\prime \prime }\left[k\right]|$ in dB; each star symbol represents the value of this equation in a certain NP region.

It is observed that DAC mismatch increases IBN power when more than two DAC levels are used for the same input level. Assuming a unit element DAC, we can consider the model depicted in Figure 7. $\vec{Q_{out}}$ is a vector that contains $2^{Nbits}-1$ elements corresponding to the quantizer output signals (thermometer coded using ±1) in each sample and $\vec{\epsilon_{DAC}}$ is a $2^{Nbits}-1$ elements vector with zero mean that contains the static deviations from nominal values of each DAC cell. $\vec{DAC_{ideal}}$ is a $2^{Nbits}-1$ elements vector that contains the nominal weights of each DAC cell. $DAC_{out}$ can take $2^{Nbits}$ different levels, as defined in (9). Three consecutive DAC levels are used in an NP region. We can define two slopes connecting these three DAC levels. The derivative of (9) is shown in (10), while the second derivative of (9) has been computed in (11). Equation (11) represents the difference between the two slopes that connect three DAC levels. If the value of (11) for a certain NP region is 0, the DAC is linear. DAC linearity in an NP region is deteriorated when the absolute value of (11) is increased. Hence, this second derivative is an estimator of linearity in NP regions. Figure 8 shows the comparison between the absolute value of (11) in dB and IBN power of a ΣΔM; there is a constant shift of 27 dB between the two magnitudes. This shift depends on modulator design parameters, as described later.

(9) $$\begin{array}{c} DAC_{levels}\left[k\right]=-\sum_{i=k}^{2^{Nbits}-1}\left(DAC_{ideal}\left[i\right]+\epsilon_{DAC}\left[i\right]\right)+\sum_{i=1}^{k-1}\left(DAC_{ideal}\left[i\right]+\epsilon_{DAC}\left[i\right]\right),\;\\ k=1,2,\ldots ,2^{Nbits} \end{array}$$

(10) $$DAC_{levels}^{\prime }\left[k\right]=2\left(DAC_{ideal}\left[k\right]+\epsilon_{DAC}\left[k-1\right]\right),\;\;\;k=1,2,\ldots ,2^{Nbits}-1$$

(11) $$DAC_{levels}^{\prime \prime }\left[k\right]=2\left(\epsilon_{DAC}\left[k+1\right]-\epsilon_{DAC}\left[k\right]\right)=2\epsilon_{DAC}^{\prime }\left[k+1\right],\;\;\;k=1,2,\ldots ,2^{Nbits}-2.$$

**Figure 9:**
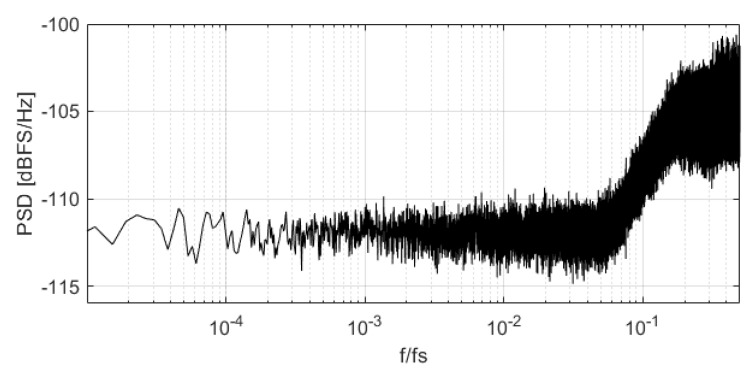
PSD of DAC mismatch error when input signal is in a NP region.

Note that each *k* NP region will have a different IBN power because the involved DAC cells have different errors. Assuming a constant mismatch for a defined CMOS technology node, the relative error of the DAC cells when compared with the full-scale range of the ΣΔM is reduced if Nbits is increased. On the other hand, NTF and OSR also impact IBN power in NP regions. Aggressive NTFs introduce more noise power and create wider NP regions than conservative NTFs due to the increased probability of using three different DAC levels, as shown in Figure 5. The PSD of the DAC error sequence ($e_{DAC}$) is flat for low and medium frequencies; this can be observed in Figure 9. If OSR is increased, then a bigger part of $e_{DAC}$ falls out of the ADC bandwidth, reducing IBN power. Modulator order is not considered because the NTF effect is properly characterized by its OBG and OSR.

#### 3.1.1. IBN Power Estimation Due to DAC Mismatch

**Figure 10:**
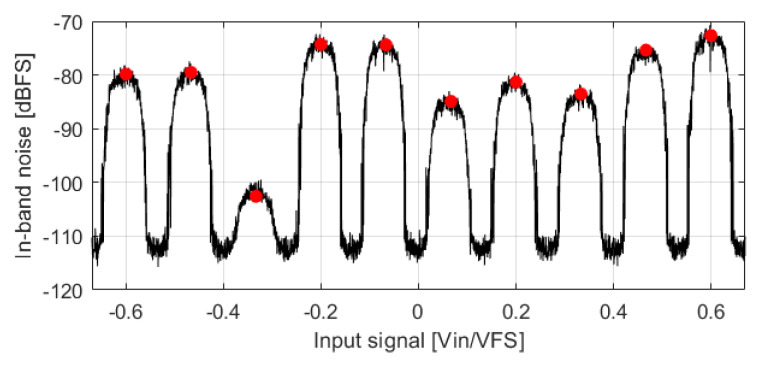
Comparison of simulated (black) and predicted (red circles) IBN power in NP regions when the DAC cells errors are known. ΣΔM parameters are: fourth-order, 4-bits, OSR=32, OBG=1.8. Output signal is divided in segments of 3.2 ms.

**Figure 11:**
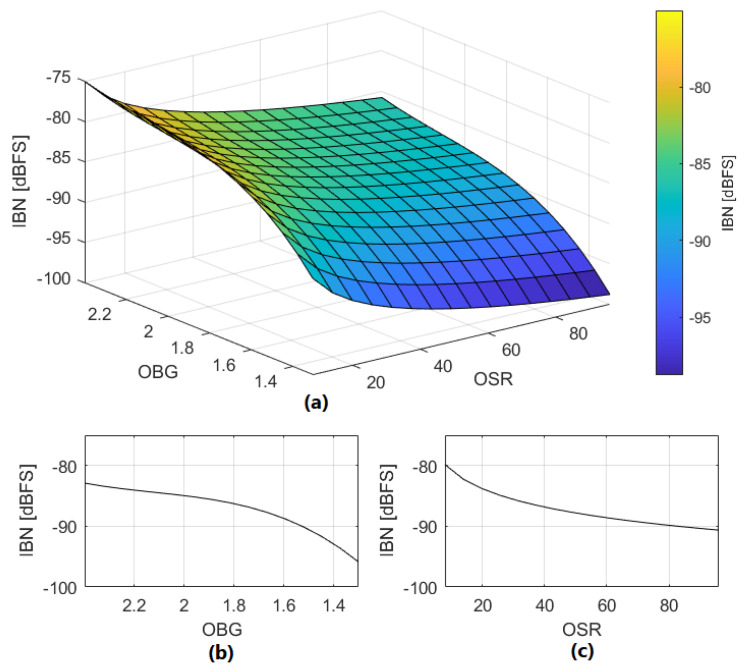
(**a**) IBN_DAC_ dependence on OSR and OBG; $|DAC_{levels}^{\prime \prime }\left[k\right]|=0.001$. (**b**) IBN_DAC_ dependence on OBG (OSR=64). (**c**) IBN_DAC_ dependence on OSR (OBG=1.8).

We propose (12) and (13) to estimate IBN power in dBFS in NP regions due to DAC mismatch. These equations model the noise behavior observed in extensive simulations. Equation (13) is valid for OBG values in the range of 1.3 to 2.4. The influence of OBG on IBN power has been obtained from multiple simulations. The results extracted from the simulations have been fitted to the polynomial expression shown in (13). An application example of this method is shown in Figure 10, estimating IBN power in NP regions with high precision if DAC cells errors are known. It is observed that DAC linearity in NP regions (11) is the most influential factor. Figure 11 shows the value of (12) for $|DAC_{levels}^{\prime \prime }\left[k\right]|=0.001$ for different OSR and OBG values.

(12) $$IBN_{DAC}\left[k\right]=20\log_{10}(|DAC_{levels}^{\prime \prime }\left[k\right]\left|\right)-10\log_{10}\left(OSR\right)-OBG_{DAC}$$

(13) $$OBG_{DAC}=152.5-196.3\cdot OBG+90.44\cdot OBG^{2}-14.2\cdot OBG^{3}.$$

Since $|DAC_{levels}^{\prime \prime }\left[k\right]|$ is different for each NP region and fabricated circuit, we derived a stochastic estimation method that allows us to obtain the probability density function (PDF) of $IBN_{DAC}$ in NP regions for a certain ΣΔM. DAC mismatch is assumed to have a normal distribution with zero mean and known standard deviation [1] defined by the design and technology ($\sigma_{T}$). To obtain the PDF of $|DAC_{levels}^{\prime \prime }|$, we first need to obtain the PDF of $\epsilon_{DAC}$:

(14) $$p\left(\epsilon_{DAC}\right)=\frac{1}{\sigma_{\epsilon }\sqrt{2\pi }}\;exp\left(-\frac{1}{2}{\left(\frac{\epsilon_{DAC}}{\sigma_{\epsilon }}\right)}^{2}\right),-\infty <\epsilon_{DAC}<+\infty$$

(15) $$\sigma_{\epsilon }=\frac{\sigma_{T}}{2^{Nbits}-1}.$$

The next step is to obtain the PDF of $\epsilon_{DAC}^{\prime }$, whose standard deviation is $\sqrt{2}\sigma_{\epsilon }$ and mean is 0. Notice that $2\epsilon_{DAC}^{\prime }=DAC_{levels}^{\prime \prime }$. The standard deviation of $DAC_{levels}^{\prime \prime }$ is $2\sqrt{2}\sigma_{\epsilon }$ and the mean is 0. The PDF of $|DAC_{levels}^{\prime \prime }|$ is:

(16) $$p(|DAC_{levels}^{\prime \prime }\left|\right)=\frac{2}{\sigma_{|DAC_{levels}^{\prime \prime }|}\sqrt{2\pi }}\;exp\left(-\frac{1}{2}{\left(\frac{|DAC_{levels}^{\prime \prime }|}{\sigma_{|DAC_{levels}^{\prime \prime }|}}\right)}^{2}\right),\left|DAC_{levels}^{\prime \prime }\right|\geq 0$$

(17) $$\sigma_{|DAC_{levels}^{\prime \prime }|}=\frac{2\sqrt{2}\;\sigma_{T}}{2^{Nbits}-1}.$$

As (16) is expressed in natural units, we need to convert the term of (12) related to OBG from logarithmic to natural; the term related to OSR is also included in this expression:

(18) $$\alpha =10^{\left(OBG_{DAC}+10\log_{10}\left(OSR\right)\right)/20}.$$

Finally, we obtain the PDF of $IBN_{DAC}$ in natural units:

(19) $$p\left(IBN_{DAC}\right)=\frac{2\alpha }{\sigma_{IBN}\sqrt{2\pi }}\;exp\left(-\frac{1}{2}{\left(\frac{IBN_{DAC}\;\alpha }{\sigma_{IBN}}\right)}^{2}\right),IBN_{DAC}\geq 0$$

(20) $$\sigma_{IBN}=\frac{2\sqrt{2}\;\sigma_{T}}{2^{Nbits}-1}.$$

Noise is usually analyzed using logarithmic units. Equation (19) is converted from natural to logarithmic for practical reasons through a variable change procedure:

(21) $$\begin{array}{c} p\left(IBN_{DAC\;dBFS}\right)=\frac{10^{\left(IBN_{DAC\;dBFS}/20-1\right)}\alpha \ln\left(10\right)}{\sigma_{IBN}\sqrt{2\pi }}exp\left(-\frac{1}{2}{\left(\frac{10^{\left(IBN_{DAC\;dBFS}/20\right)}\alpha }{\sigma_{IBN}}\right)}^{2}\right),\;\\ -\infty <IBN_{DAC\;dBFS}<+\infty \end{array}$$

**Figure 12:**
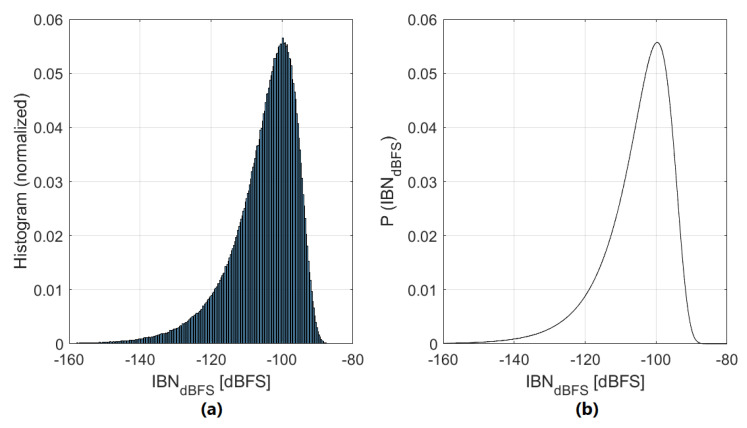
Comparison of (21) with 100,000 simulations of noise power in NP regions. ΣΔM parameters are: third-order, 4-bits, OSR=38, OBG=1.8, and σT=0.1%. (**a**) Normalized histogram. (**b**) Equation (21).

Figure 12 compares (21) with multiple simulations of IBN power in NP regions. It is clear that the equation perfectly matches the simulation.

**Figure 13:**
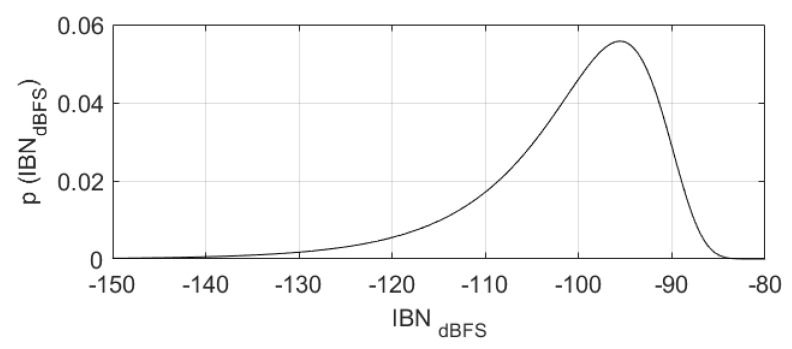
IBN power in NP regions PDF (in dBFS) for a third-order ΣΔM with 4-bits, fs=2.32 MHz, BW=20 kHz, OSR=58, OBG=1.8, and σT=0.2%.

A third-order, 4-bits, fs=2.32 MHz, BW=20 kHz, OSR=58, OBG=1.8ΣΔM-ADC with high resolution for audio applications generated with the Schreier toolbox is used as an example to show the effect of IBN power increase due to DAC mismatch. The PDF of IBN power in NP regions in dBFS assuming $\sigma_{T}=0.2\%$ is shown in Figure 13, where (21) has been plotted. Quantization noise is designed to be −119 dBFS; for a power efficient design, thermal noise should be 12 dB higher (−107 dBFS) [1]. $IBN_{DAC}$ power is mainly distributed between −150 dBFS and −85 dBFS, meaning that the noise power increase can potentially be 22 dB higher than thermal noise. The probability of a certain NP region being above the thermal noise level is 96.5%; the probability of being 12 dB higher than the thermal noise level is 56.5%, showing that IBN power increase is very likely to degrade the ADC performance. The probability of having at least one NP region with a noise power increase 12 dB higher than the thermal noise is higher than 99.99%, meaning that almost all manufactured circuits will be affected by the noise power increase.

#### 3.1.2. Dynamic Element Matching

**Figure 14:**
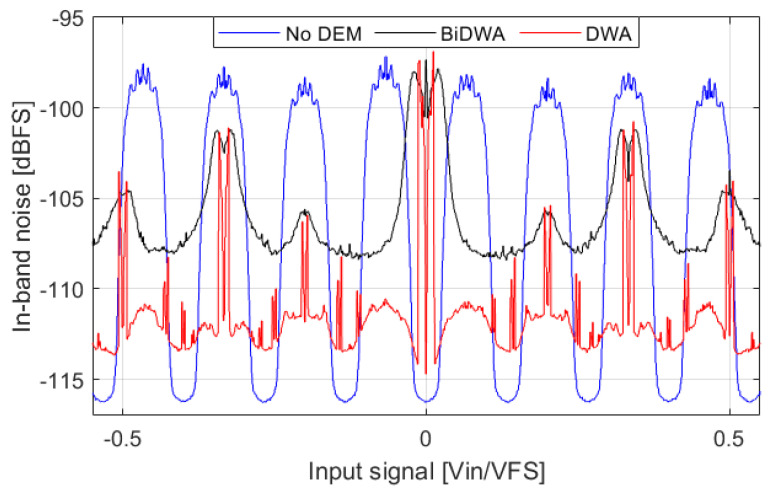
IBN power comparison for the following cases: no DEM, DWA, and Bi−DWA. In total, 300 simulations have been averaged. Input signal is a slow ramp. Output signal is divided in segments of 2.69 ms. ΣΔM parameters are: fourth-order, 4-bits, fs=1.52 MHz, BW=20 kHz, OSR=38, OBG=1.8, $\sigma_{T}$ = 0.2%.

The error introduced by DAC mismatch in multi-bit ΣΔMs is typically shaped using Dynamic Element Matching (DEM) techniques. Data Weighted Averaging (DWA) [15] and Bi-Directional Data Weighted Averaging (Bi-DWA) [16] have been tested to check if the observed noise in NP regions can be reduced. Both DWA and Bi-DWA achieve first-order mismatch shaping; Bi-DWA also eliminates tones at the cost of increasing the noise floor. Figure 14 shows the IBN power comparison for the following cases: no DEM, DWA, and Bi-DWA. A total of 300 simulations have been averaged for each case to acknowledge the statistical behavior of the DAC mismatch. The modulator parameters are equal. DWA and Bi-DWA increase IBN power outside of NP regions by 3 dB and 9 dB, respectively (excepting the zero crossing region). IBN power in NP regions is reduced by both DWA and Bi-DWA, with DWA being the most effective in reducing noise power and NP regions width. The most important finding is that the simulated DEM techniques create a noise bump when the amplitude of the input signal is close to zero. This happens because these DEM techniques create a repetitive pattern for input signals close to zero, not being able to shape the mismatch noise. On top of that, this behavior increases $e_{DAC}$ because all the DAC cells are constantly toggling. If the input signal is a low level (−40 dBFS) sine-wave without DC component, DWA, and Bi-DWA increase the noise power by 17 dB and 18 dB, respectively.

### 3.2. The Effect of Loop Filter Linearity Error on IBN Power Increase

**Figure 15:**
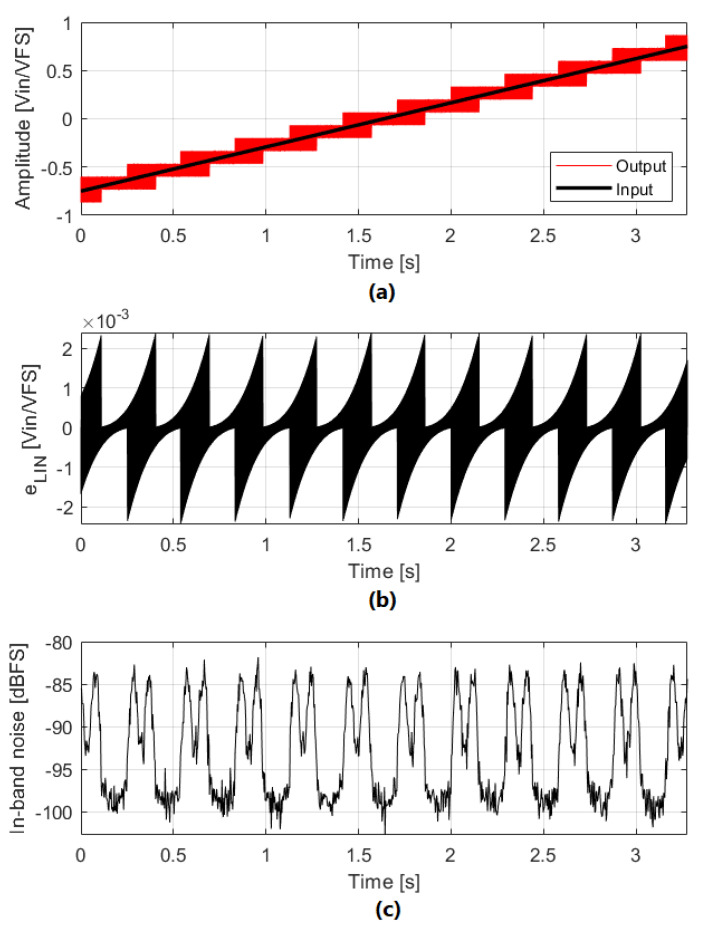
IBN power dependence on the input signal due to loop filter linearity error. ΣΔM parameters are: third-order, 4-bits, OSR=32, OBG=1.8, $g_{3}=0.5$. (**a**) Input and output signals. (**b**) Loop filter linearity error. (**c**) In-band noise power. Output signal is divided in segments of 23.2 ms.

Loop filter linearity is usually governed by the linearity of the first integrator amplifier; the rest of the amplifiers have reduced impact because their errors are shaped by the loop gain. The linearity of an amplifier depends on the signal level at its input. When the modulator input signal is close to a quantizer threshold, the ΣΔM operates toggling between two different levels. The loop filter input signal is concentrated around $\pm V_{LSB}/2$; if the amplifier is properly designed, linearity should not be a problem. If the modulator input signal is in an NP region, the ΣΔM toggles using three different levels; the loop filter input signal is concentrated around $\pm V_{LSB}$ and 0, causing a bigger linearity error. IBN power increase due to loop filter linearity error is equal in all the NP regions for a given ΣΔM. This behavior can be observed in Figure 15.

The linearity error introduced depends on the amplifier architecture; if fully differential OTAs are used, the amplifier transfer function can be modeled as the combination of a linear component and a third-order non-linear component ($g_{3}$) [1]. The non-linear component $g_{3}$ can be extracted from circuit simulations. Amplifier linearity error when the loop filter input signal is $\pm V_{LSB}$ can be obtained as:

(22) $$\epsilon_{AMP}=g_{3}\cdot {\left(\pm V_{LSB}\right)}^{3}$$

**Figure 16:**
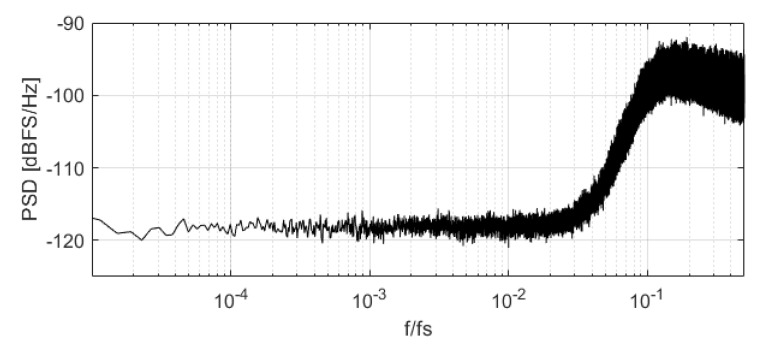
PSD of the loop filter linearity error when the input signal is in a NP region.

The OBG determines the probability of generating a value close to $\pm V_{LSB}$ at the loop filter input; IBN power in NP regions is increased if high OBG values are used. If OSR is increased, a bigger part of $\epsilon_{AMP}$ falls out of the bandwidth, reducing IBN power because the PSD of $\epsilon_{AMP}$ is flat for low and medium frequencies (shown in Figure 16). When the number of bits of the quantizer is increased, $V_{LSB}$ is decreased and consequently $\epsilon_{AMP}$ and IBN power are reduced too.

#### IBN Power Estimation Due to Loop Filter Linearity Error

**Figure 17:**
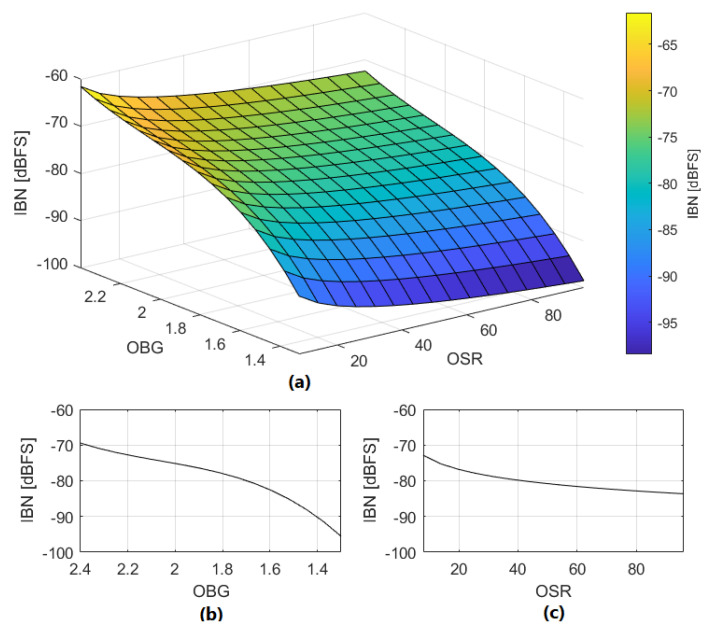
(**a**) IBN_AMP_ dependence on OSR and OBG; $|\epsilon_{AMP}|=0.003$. (**b**) IBN_AMP_ dependence on OBG (OSR=64). (**c**) IBN_AMP_ dependence on OSR (OBG=1.8).

The proposed estimation method is based on the observations extracted from extensive simulations. Equations (23) and (24) are used to estimate IBN power in dBFS in NP regions due to loop filter linearity error. Equation (24) is valid for OBG values in the range of 1.3 to 2.4. The influence of OBG on IBN power has been obtained from multiple simulations. The results extracted from these simulations have been fitted to the polynomial expression shown in (24). Figure 17 shows the value of (23) for $|\epsilon_{AMP}|=0.003$ for different OSR and OBG values.

(23) $$IBN_{AMP}=20\log_{10}\left(\right|\epsilon_{AMP}\left|\right)-10\log_{10}\left(OSR\right)-OBG_{AMP}$$

(24) $$OBG_{AMP}=274.65-366.15\cdot OBG+171.94\cdot OBG^{2}-27.79\cdot OBG^{3}$$

This prediction method has been tested in different ΣΔMs, showing good agreement between estimations and simulation results, as can be seen in Table 1. The estimation error is lower than 3 dB for all the simulated cases. $\epsilon_{AMP}$ is expressed as a percentage of $V_{LSB}$ to show that even very low relative errors have a noticeable impact. It is observed that the most important factor in determining $IBN_{AMP}$ is the term related to the amplifier linearity error (22). Modulator order has not been included in the proposed estimation method because the NTF effect is properly characterized by its OBG and OSR.

### 3.3. The Effect of Quantization Error on IBN Power Increase

**Figure 18:**
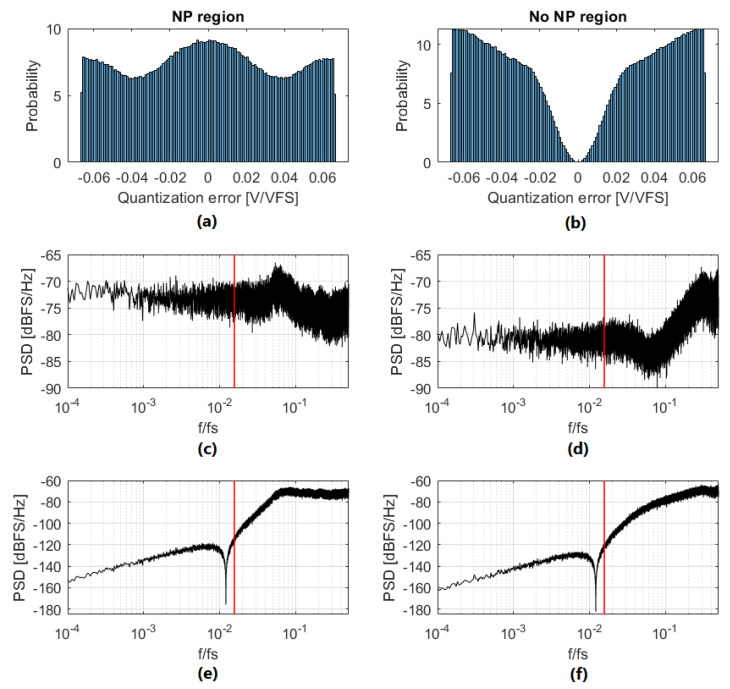
Quantization error comparison (in and outside of NP regions). ΣΔM parameters are: third-order, 4-bits, OSR=32, OBG=1.5. Histogram of quantization error in NP regions (**a**) and outside of NP regions (**b**). PSD of quantization error in NP regions (**c**) and outside of NP regions (**d**); vertical red line marks the modulator bandwidth. PSD of quantization error at the ΣΔM output in NP regions (**e**) and outside of NP regions (**f**); vertical red line marks the modulator bandwidth.

Quantization error in ΣΔMs is usually assumed to be an additive white noise sequence, independent of the quantizer input and uniformly distributed between $-V_{LSB}/2$ and $+V_{LSB}/2$ [17]. If the real behavior of a ΣΔM is analyzed, it can be seen that the quantization error sequence depends on the modulator input signal, it is not white, and it is not uniformly distributed. The statistical and spectral properties of the quantization noise sequence vary depending on the input signal level. If the input signal is at low frequencies, it will cross NP regions, and regions in which only two different levels are used. A comparison of quantization error, in and outside of NP regions, can be observed in Figure 18. The histograms (a) and (b) clearly show that the quantization error PDF depends on the input signal level, being extremely different in NP regions and outside of NP regions. The histograms do not have a uniform distribution for any of the simulated cases. Figure 18c,d show the PSD comparison in and outside of NP regions. IBN power is −36.51 dBFS in NP regions, and −45.47 dBFS outside of NP regions. Quantization IBN power is increased by 8.964 dB in NP regions.

**Figure 19:**
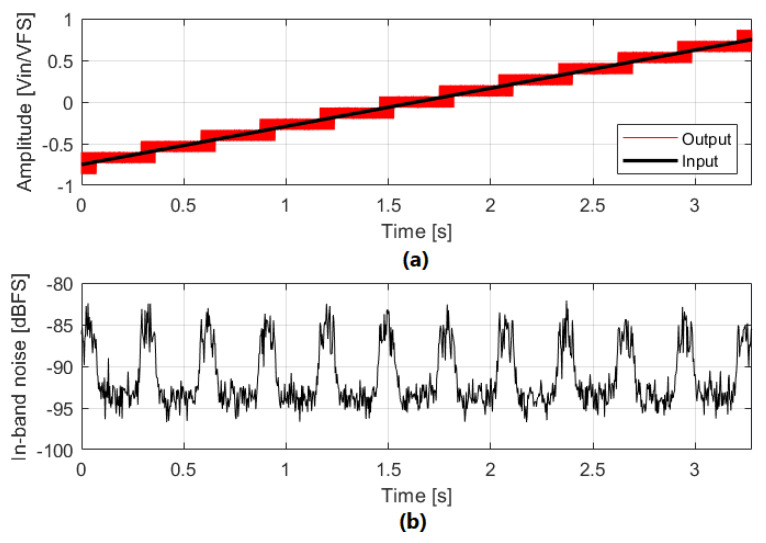
IBN power dependence on the number of levels due to quantization error. ΣΔM parameters are: third-order, 4-bits, OSR=32, OBG=1.5. (**a**) Input and output signals. (**b**) In−band noise power. Output signal is divided in segments of 2.13 ms.

The quantization error shown in Figure 18c,d appears at the ΣΔM output filtered by the NTF, as shown in Figure 18e,f. The filtering operation reduces IBN power from −36.51 dBFS to −86.50 dBFS in NP regions and from −45.47 dBFS to −95.36 dBFS outside of NP regions. As in the previous case, IBN power at the ΣΔM output is increased by 8.96 dB in NP regions. This leads to increased IBN power at the output of the ΣΔM when the input signal is in an NP region, as can be seen in Figure 19. IBN power is equal for all the NP regions.

The influence of the ΣΔM parameters on IBN power increase in NP regions due to quantization error ($\Delta IBN_{Q}$) has been analyzed in Table 2. Each modulator parameter has been varied individually to observe its influence. Modulator order, OSR and Nbits have negligible effect on IBN power increase. If they are modified, the maximum variation of $\Delta IBN_{Q}$ is 1 dB; this is negligible when compared to the influence of OBG. It is clear that OBG determines $\Delta IBN_{Q}$. The smaller OBG, the bigger $\Delta IBN_{Q}$. This case is not common because multi-bit operation in ΣΔMs allows the use of higher OBG values without compromising stability, thereby achieving better noise shaping. On top of that, thermal noise is usually designed to be 12 dB higher than quantization noise power in two level regions [1]. This means that an IBN power increase due to quantization error is very unlikely to have any effect on the total IBN power.

#### IBN Power Estimation Due to Quantization Error

**Figure 20:**
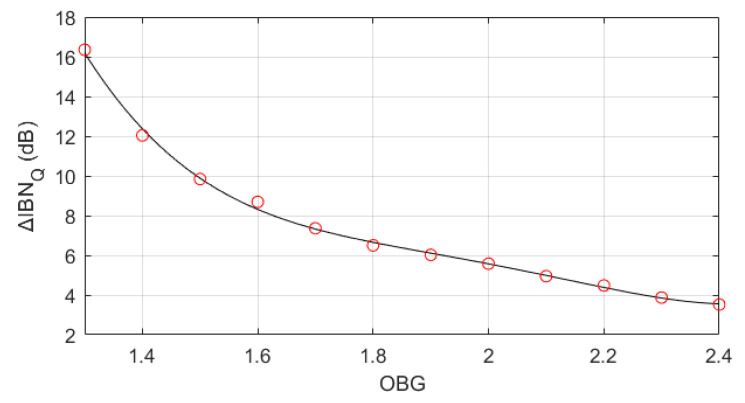
Dependence of $\Delta IBN_{Q}$ on NTF OBG. Red circles show simulation results; a black line shows the estimation using (25).

From the simulation results of Table 2, we conclude that the effect of OSR and ΣΔM order on $\Delta IBN_{Q}$ is negligible, when compared to the effect of OBG. Equation (25) can be used to estimate $\Delta IBN_{Q}$; it has been obtained through a polynomial regression based on simulation results (Figure 20). $\Delta IBN_{Q}$ has exponential behavior for OBG<1.7, reaching values as high as 16 dB. Nevertheless, this case is very unlikely because quantization noise shaping is not optimized. $\Delta IBN_{Q}$ has linear behavior for OBG≥1.7, having low values when compared with the thermal noise level (that should be 12 dB higher than quantization noise outside of NP regions). Theoretical IBN power outside of NP regions can be obtained using (26) [1], where OSR and modulator order have a great impact as expected. IBN power in NP regions (in dBFS) can be estimated using (27).

(25) $$\Delta IBN_{Q}=502.88-979.01\cdot OBG+728.43\cdot OBG^{2}-241.43\cdot OBG^{3}+29.9024\cdot OBG^{4}$$

(26) $$IBN_{NAT}=\frac{{V_{LSB}}^{2}}{12\pi }\int_{0}^{\frac{\pi }{OSR}}{\left|NTF(\omega )\right|}^{2}d\omega$$

(27) $$IBN=10\log_{10}\left(IBN_{NAT}\right)+\Delta IBN_{Q}.$$

## 4. Discussion

All the noise sources analyzed in this article are combined in practical applications. The target of this section is to discuss the influence of each noise source on total IBN power. $e_{DAC}$ is strongly dependent on DAC mismatch ($\epsilon_{DAC}$, determined by $\sigma_{T}$). $e_{LIN}$ relies on first amplifier linearity ($\epsilon_{AMP}$, determined by $V_{LSB}$ and $g_{3}$). Quantization IBN power in NP regions is mainly determined by the OBG of NTF, and is not likely to be higher than thermal noise power, as explained in Section 3.3; therefore, the influence of $e_{Q}$ is very reduced in practical applications. Regarding $e_{DAC}$ and $e_{LIN}$, there is no general rule to determine which noise source is dominant. Each ΣΔM design must be analyzed individually, taking into account all system parameters (NTF, OSR, Nbits) and circuit parameters ($g_{3}$, $\sigma_{T}$). Table 3 can be used as a design guide in the case that a ΣΔM designer detects IBN power higher than expected in NP regions. The dominant noise source must be identified, and then one or multiple of the suggested parameters should be modified.

**Figure 21:**
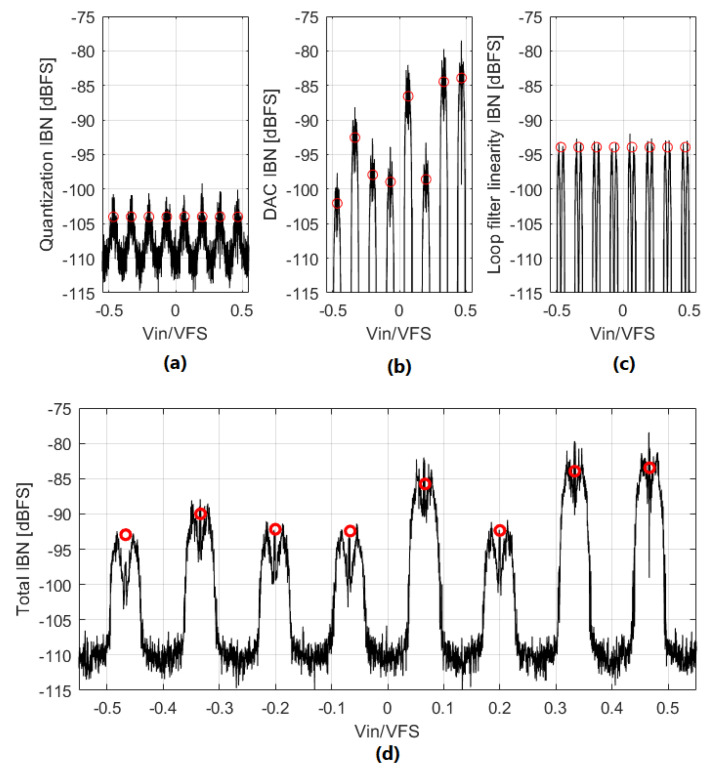
Comparison of the contributions of the different IBN sources analyzed in this article. The black lines represent the simulated IBN power; the red circles mark the estimated IBN power in NP regions. Input signal is a very slow ramp. ΣΔM parameters are: third-order, 4-bits, OBG=1.7, OSR=48, g3=0.25. Output signal is divided in segments of 2.13 ms. (**a**) Quantization IBN power. (**b**) DAC mismatch IBN power. (**c**) Loop filter linearity error IBN power. (**d**) Total IBN power.

All the estimation methods shown in this chapter can be combined to estimate the total IBN power. Figure 21 shows the IBN power generated by each noise source, the total IBN power, and the predicted IBN power in NP regions using (12), (23) and (27). IBN power in NP regions is estimated with great accuracy for all the noise sources. It is clear that $e_{DAC}$ and $e_{LIN}$ are the dominant noise sources for this ΣΔM.

## 5. Conclusions

In this article, we have shown an analysis of the IBN power increase in NP regions in multi-bit ΣΔMs. This has been observed in ΣΔ based sensors excited with low-frequency signals of medium or high amplitude. In this situation, the modulator is forced to use more than two DAC levels for a significant period of time, and hence, shows bigger sensitivity to known error sources, as DAC mismatch. This phenomenon has been compared with idle tones produced by DC inputs. Surprisingly, the observed noise bumps are correlated with increased noise floors and are not with tonal behavior. As additional proof, we have shown how dithering the input of the ADC only worsens the performance.

Three error sources have been identified as the main contributors to the observed noise increments: DAC mismatch, loop filter linearity, and quantization error. The effect of DAC mismatch has been analyzed first, showing its dependence on the modulator parameters as well as the standard deviation of the mismatch, which depends on the circuit design and the technology node. Two estimation methods of IBN power in NP regions have been proposed. The first one assumes that the error of each cell is known. The second one is a stochastic extension of the first one that uses the well-known statistical properties of the mismatch to obtain a generalized method that provides a PDF of the IBN power in NP regions. The deviation of this estimation method is 0 dB. The effect of DEM techniques on IBN power reduction in NP regions has been shown. The loop filter linearity error effect has also been studied, showing that the main contributor is the linearity error of the first amplifier. There is a clear dependence on the modulator parameters and the linearity error of the amplifiers. An estimation method of IBN power in NP regions based on extensive simulations has been obtained, showing a maximum deviation of 3 dB. Lastly, the influence of quantization error on IBN power in NP regions has been analyzed, showing that the statistical properties of the quantization error depend on the input signal. This causes the IBN power in NP regions to be higher than outside of NP regions. The IBN power increment due to quantization error only depends on NTF OBG; this means that it will be present even in an ideal ΣΔM. An estimation method of IBN power in NP regions has been proposed showing a maximum deviation of 0.5 dB.

Finally, all the described error sources have been compared. A general rule to determine which source is dominant cannot be established; each ΣΔM design must be analyzed individually. The previously proposed IBN power estimation methods can be combined to obtain the total IBN power in NP regions, showing great accuracy.

## Figures and Tables

**Table 1:** Comparison between simulated and predicted IBN power in NP regions due to amplifier linearity error.

ΣΔM Order	NTF OBG	OSR	Nbits	$\epsilon_{\mathrm{AMP}}$ (% of $V_{\mathrm{LSB}}$)	Simulated IBN Power (dBFS)	Predicted IBN Power (dBFS)
2	1.8	64	4	4.44	−75.03	−73.21
2	2	48	5	0.20	−104.52	−102.04
2	2.2	72	4	2.66	−75.24	−72.95
2	2.3	32	5	0.20	−99.23	−96.43
3	1.3	58	3	1.22	−93.57	−94.97
3	1.5	56	4	0.53	−99.30	−96.43
3	1.8	38	4	0.44	−91.44	−91.02
3	2.4	12	5	0.20	−91.98	−90.66
4	1.3	38	3	2.15	−86.59	−88.21
4	1.8	16	3	1.63	−69.08	−68.54
4	2.1	32	4	0.53	−82.78	−84.69
4	2.25	12	4	0.26	−82.42	−84.48

**Table 2:** Influence of the ΣΔM parameters on $\Delta IBN_{Q}$.

ΣΔM Order	NTF OBG	OSR	Nbits	IBN Power Outside of NP Regions (dBFS)	IBN Power in NP Regions (dBFS)	$\Delta {\mathrm{IBN}}_{Q}$ (dB)
3	1.8	32	4	−99.23	−92.73	6.50
2	1.8	32	4	−85.51	−79.12	6.39
4	1.8	32	4	−113.51	−106.26	7.25
3	1.5	32	4	−93.65	−83.81	9.84
3	2.1	32	4	−101.62	−96.67	4.95
3	1.8	16	4	−79.07	−71.92	7.15
3	1.8	64	4	−117.57	−111.18	6.39
3	1.8	32	3	−92.38	−85.71	6.67
3	1.8	32	5	−106.25	−99.15	7.10

**Table 3:** Suggested modifications to reduce IBN power in NP regions.

Dominant Noise Source	Suggested Changes
DAC mismatch error	Increase OSR
	Decrease OBG
	Decrease $\sigma_{T}$
	Increase Nbits
Loop filter linearity error	Increase OSR
	Decrease OBG
	Decrease $g_{3}$
	Increase Nbits
Quantization error	Increase OBG

## Data Availability

No new data were created or analyzed in this study. Data sharing is not applicable to this article.
